# Uterine Hydatids

**Published:** 1854-09

**Authors:** Delos W. Young

**Affiliations:** Aurora, Illinois


### ART. II.—*Uterine Hydatids.* By Delos W. Young, M.D., Aurora, Illinois.

October 25th, 1853, I was called to see Mrs. F. aged 18 years. Has been married three months, and is laboring under an attack of leucorrhœa. She gave me the following history of her case. She first began to menstruate at the age of 13, when she was advised by her sister and other *female* friends to expose herself to cold and wet, in order to *harden her system*, assuring her that she could do so with impunity, and bear her future *turns* much better. Accordingly, she went barefooted, and stood in a tub of cold water. She took cold, which produced suppression of the menses, followed by fever of several days duration; since which time she has suffered from periodical attacks of leucorrhœa, but has never menstruated regularly.

I now found her anemic, with feeble circulation; bowels have been constipated for the past year; complaining of headache and general prostration. Leucorrhœa profuse and somewhat painful. Ordered cathartic mixture (composed of aloes, spts. lavendula comp., soda bicar. and water), astringent vaginal injections, wine and iron, with nutritious diet.

November 14.—I find my patient's general health much improved, bowels more regular. Advised continuance of tonics.

March 22d, 1854.—I was called to see Mrs. F., and found her with profuse uterine hemorrhage; abdomen slightly bloated, but yielded readily to treatment with ergot and cold applications to the abdomen.

April 8.—Had another similar attack, which was relieved by like treatment.

May 30.—Was called to see patient; had some hemorrhage; bowels bloated, and complaining of pains in the hypogastric region. Supposes herself pregnant, but utterly refuses to submit to a vaginal examination. Ordered rest in a recumbent posture, and cold applications to the abdomen.

June 18.—Had another attack of hemorrhage, and abdomen is more distended. Still refuses to submit to a vaginal examination. Treatment as before.

June 28.—Is attacked as before, with fulness of abdomen increasing.

July 13, 5 o'clock, A.M.—I was called in haste; found patient with a slight attack of hemorrhage; much agitated; suffering severe pain in the uterine region; abdomen largely distended. Has not slept during the previous night. Refuses to be examined. Prescribed anodyne, with cold applications to the abdomen and vulva, and perfect quiet.

10 o'clock, A.M.—Patient attacked suddenly with profuse hemorrhage. I being absent, my partner, Doctor Abner Hard, visited the patient, and found her cold, faint, and almost pulseless. Made vaginal examination; found os tincæ slightly dilated, so as only to admit the index finger. Could touch a soft, pulpy mass, supposed to be clots of blood, but could feel nothing like a fœtus. Owing to profusion of hemorrhage, and prostration of the patient, he prescribed brandy, camphor and ergot, introduced ice into the vagina, and applied warmth to the extremities; which, with kneading of the bowels, secured contraction of the uterus, and expelled some clots of blood.

Hemorrhage ceased, pulse began to improve. Made another examination; found os tincæ as before. Felt a substance resembling placenta, but could detect nothing like a fœtus. Continued stimulants, and despatched a messenger for me. I arrived at 11½ A.M.; found patient as above described.

Advised continuance of brandy, with addition of chloroform, and repetition of ergot, as there was a little hemorrhage.

12½ P.M.—Patient has some uterine pains, with more hemorrhage. Made examination, and removed small pieces of a substance resembling placenta.

1 o'clock P.M.—We found the os tincæ sufficiently dilated to admit two fingers, and removed a substance found to be uterine hydatids. Contraction continued regularly, until we succeeded in removing about five quarts of hydatids, resembling in form clusters of grapes, a large quantity of which we have preserved in alcohol.

After treatment, applied bandage round the body; ordered cold to the vulva and abdomen, with perfect quiet in the recumbent posture.

July 14.—9 o'clock A.M.—Patient rested well; no hemorrhage, and convalesced gradually; up to this time is doing well.

*Query*.—How long have the hydatids been growing? Was their growth the cause of leucorrhœa, and consequent ill health? or was the leucorrhœa and copulation the cause of their growth? Editors, please give your opinion.

